# Antioxidant Therapy for Atrial Fibrillation: What Is the Next Step?

**DOI:** 10.4061/2011/429537

**Published:** 2011-11-03

**Authors:** Ali A. Sovari

**Affiliations:** Section of Cardiology, Center for Cardiovascular Research, University of Illinois at Chicago, 840 S. Wood Street, MC 715, Chicago, IL 60612, USA

I read with interest the timely article by Rasoli and colleagues, who reviewed the literature on the efficacy of antioxidant vitamins in the management of atrial fibrillation (AF) [1]. Those authors emphasized an important clinical issue: despite considerable evidence of the role of oxidative stress in the pathogenesis of AF, antioxidant vitamins have not been shown to exert an impressive antiarrhythmic effect in clinical trials. In addition to the authors' explanations for that finding, it may also be possible that treatment with a general reactive oxygen species (ROS) scavenger is ineffective, regardless of the type or potency of the scavenger. This may occur because ROS are a wide range of (usually) highly active molecules that, when they are formed, react with proteins and lipids in their immediate vicinity. Therefore, ROS scavengers cannot prevent the effects of already formed ROS molecules. In addition, those scavengers may not neutralize all forms of ROS molecules, some of which may remain to exert a downstream arrhythmic effect. 

Inhibiting the major source of excess ROS is a different therapeutic approach that may prove effective in the management of various types of arrhythmia. The major sources of cardiac ROS are nicotinamide adenine dinucleotide phosphate (NADPH) oxidase, the mitochondria electron transport chain, and uncoupled endothelial nitric oxide synthase. Those sources of ROS are interrelated, and the activation of one may result in the increased production of ROS by other sources, particularly by mitochondria (ROS-induced-ROS release) [2]. Under different conditions and for each type of AF, one or some of the main sources of ROS may play the central role in the genesis of arrhythmia. It has been shown that NADPH oxidase is upregulated in initial stages of AF [3], and therefore, NADPH oxidase inhibitors may be effective in the management of new onset AF and postoperative AF [4]. However, the upregulation of NADPH oxidase was found to be transient and only in initial stages of AF, and therefore, its inhibition may not be an effective therapeutic strategy for the management of chronic AF [3]. Because a considerable portion of a cardiomyocyte is occupied by mitochondria [5] and because mitochondria plays a central role in ROS-induced-ROS release, mitochondria-targeted antioxidant therapy may prove to be the most effective antioxidant therapy for management of chronic AF. We have recently observed the impressive antiarrhythmic effect of Mito-TEMPO, a mitochondria-targeted antioxidant, in the prevention of ventricular arrhythmias in a mouse model of angiotensin-II activation [6]. Thus, it could be hypothesized that mitochondria-targeted antioxidants can effectively prevent chronic AF.
